# The Effect of Ten Essential Oils on Several Cutaneous Drug-Resistant Microorganisms and Their Cyto/Genotoxic and Antioxidant Properties

**DOI:** 10.3390/molecules24244570

**Published:** 2019-12-13

**Authors:** Katarína Kozics, Mária Bučková, Andrea Puškárová, Viktória Kalászová, Terézia Cabicarová, Domenico Pangallo

**Affiliations:** 1 Cancer Research Institute BMC, Slovak Academy of Sciences, Dúbravská cesta 9, 845 05 Bratislava, Slovakia; katarina.kozics@savba.sk; 2 Institute of Molecular Biology, Slovak Academy of Sciences, Dúbravská cesta 21, 84551 Bratislava, Slovakia; maria.buckova@savba.sk (M.B.); andrea.puskarova@savba.sk (A.P.); 3 Department of Genetics, Faculty of Natural Sciences, Comenius, University, Mlynská dolina, 842 15 Bratislava, Slovakia; viktoria.kalaszova@gmail.com; 4 Food Research Institute, National Agricultural and Food Centre, Priemyselná 4, 824 75 Bratislava, Slovakia; tereza.cabicarova@nppc.sk

**Keywords:** multi-drug-resistant bacteria, drug-resistant *Candida*, essential oils, cyto/genotoxic effects, total antioxidant status, human keratinocytes HaCaT

## Abstract

In this study, we determined the antimicrobial activity of ten essential oils (EOs)—oregano, thyme, clove, arborvitae, cassia, lemongrass, melaleuca, eucalyptus, lavender, and clary sage—against drug-resistant microorganisms previously isolated from patients with skin infections. The essential oil compositions were determined using gas chromatography coupled to mass spectrometry (GC/MS). The assayed bacteria included *Pseudomonas aeruginosa*, *Proteus vulgaris, Citrobacter koseri,* and *Klebsiella pneumoniae*. Two drug-resistant yeasts (*Candida* *albicans* and *Candida parapsilosis*) were also involved in our survey. Oregano, thyme, cassia, lemongrass and arborvitae showed very strong antibacterial and antifungal activity against all tested strains. These results show that these essential oils may be effective in preventing the growth of the drug-resistant microorganisms responsible for wound infections. In this study, the genotoxic effects of tested essential oils on healthy human keratinocytes HaCaT were evaluated using the comet assay for the first time. These results revealed that none of the essential oils induced significant DNA damage in vitro after 24 h. Moreover, the treatment of HaCaT cells with essential oils increased the total antioxidant status (TAS) level. The obtained results indicate that EOs could be used as a potential source of safe and potent natural antimicrobial and antioxidant agents in the pharmaceutical and food industries.

## 1. Introduction

Human skin is permanently exposed to its external environment, and this makes skin issues among the most common infections in the world. Dermatitides are one of the five most frequently treated medical problems [1,2]. Medical treatments and healing may be influenced by the presence of pathogenic microorganisms. Available treatments are often inefficient, due to infections with drug-resistant microorganisms such as *Pseudomonas aeruginosa, *Klebsiella pneumoniae,* Citrobacter freundii*, *Proteus vulgaris*, *Acinetobacter baumanii, Staphylococcus aureus* and *Candida* sp. [3,4,5]. During the past several decades, the occurrence of multiresistant bacteria and fungi poses a serious problem worldwide, making choosing the appropriate treatment for patients affected with skin infections a challenge [6,7].

Natural products, especially essential oils (EOs), have been suggested as antimicrobial agents [8,9]. EOs are known to possess antimicrobial properties against multiresistant bacteria and fungi, due to a broad spectrum of biocidal activity [10,11]. EOs are volatile compounds produced by the secondary metabolism of plants, and are mainly composed of terpenoids, terpenes, and aliphatic and aromatic constituents [12]. Generally, essential oils characterized by a high level of phenolic compounds, such as carvacrol, eugenol, and thymol, have important antibacterial and antifungal properties [13,14,15]. EOs from eucalyptus (EU), clove (CL), oregano (OR), thyme (TY), clary sage (SA) lavender (LA) and Cassia (CA) demonstrated various antibacterial, antifungal, antiseptic, antitumor, antihyperglycemic, antioxidant and anti-inflammatory effects [16,17,18,19]. The EOs of lemongrass (LE) and arborvitae (AR) have been tested for antimicrobial [11,16,20] and insecticidal abilities [21].

Skin is the human body’s primary barrier to the environment, and the first line of defense against microbial and chemical attacks [22]. Microbial infection is the most dangerous consequence of skin injury [23,24,25]. High concentrations of EOs in products designed for treatment of skin problems (e.g., acne or mycosis) might be toxic to epithelial cells [26]. Therefore, the determination of the cyto/genotoxic and antioxidant effects of EOs might greatly improve their application in the treatment of skin problems.

In this report, we have screened the antimicrobial properties of 10 essential oils against multi-drug-resistant (MDR) bacteria, namely *Pseudomonas aeruginosa*, *Proteus vulgaris, Citrobacter koseri, Klebsiella pneumoniae*, and drug-resistant (DR) *Candida albicans* and *Candida parapsilosis* (two isolates), isolated from patients with skin infections. In addition, the EOs were also screened for cyto/genotoxic properties and their total antioxidant level in an “in vitro” model using normal human keratinocytes HaCaT.

## 2. Results

### 2.1. EOs Composition

As depicted in Table 1, EOs were chosen according to their chemical composition, particularly for their more abundant components (Figure S1). The major compounds of the EOs cassia and lemongrass were aldehydes. Oregano, thyme (thymol chemotype) and clove mainly contained phenolic derivatives. In melaleuca and lavender, terpene alcohols were identified. The major compounds in eucalyptus were oxides, and in clary sage esters were detected. The essential oil of arborvitae mainly contained a monoterpene backbone with an ester functional group. The retention indexes of EOs are shown in Table S1.

### 2.2. Antimicrobial Susceptibility Testing to Antibiotics and Antimycotics

The resistance to antibiotics or antimycotics of the target microorganisms (four clinical bacterial strains: *K. pneumoniae*, *P. aeruginosa*, *P. vulgaris*, *C. koseri*, and three yeast strains *C. albicans*, *C. parapsilosis*) is shown in Table 2 and Table 3. The results show that the strains isolated from wound infections were highly resistant to most of the β-lactam, cephalosporin, aminoglycoside and quinolone antibiotics, and also to the azoles.

### 2.3. Screening of EOs Antimicrobial Ability

**Figure 1:**
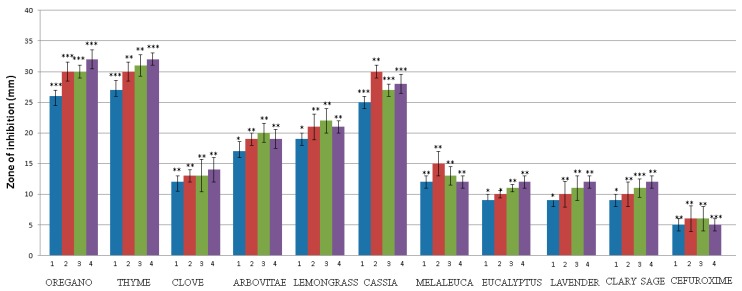
Antibacterial activity of assayed essential oils. Cefuroxime (30 μg/disc) was used as a positive control for bacterial inhibition. Each bar of the chart shows the mean diameter of the inhibition halos obtained for each essential oil (EO) analyzed; (1) *P. aeruginosa* KMB527, (2) *P. vulgaris* KMB525, (3) *K. pneumoniae* KMB522, (4) *C. koseri* KMB526. Data are represented by means ± 1 SD of three independent experiments. * *p* < 0.05; ** *p* < 0.01; *** *p* < 0.001 indicate statistically significant differences compared to the control (Student’s t-test).

**Figure 2:**
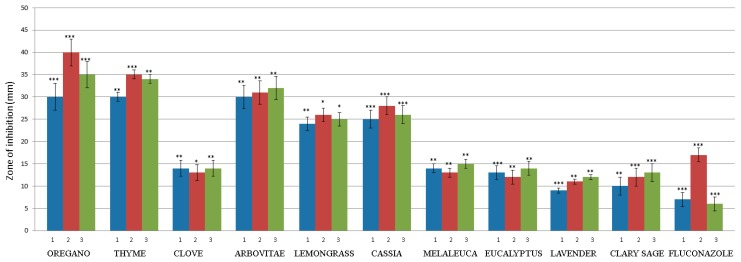
Antifungal activity of assayed essential oils. Fluconazole (20 μg/disc) was used as a positive control for fungal inhibition. Each bar of the chart shows the mean diameter of the inhibition halos obtained for each EO analyzed; (1) *C. albicans* Nr. 2, (2) *C. parapsilosis* Nr. 8, (3) *C. parapsilosis* Nr. 52. Data are represented by means ± 1 SD of three independent experiments. * *p* < 0.05; ** *p* < 0.01; *** *p* < 0.001 indicate statistically significant differences compared to the control (Student’s t-test).

The antimicrobial activity of the EOs on the growth of MDR bacteria and drug-resistant *Candida* species was assessed using the agar disc diffusion assay. The results obtained for EO are presented in Figure 1 and Figure 2. The growth of MDR bacteria and drug-resistant *Candida* species was inhibited by the presence of 10 µL of the oil at a concentration of 100%. A broad variation in antimicrobial properties of the analyzed EOs was observed in the study (* *p* < 0.05; ** *p* < 0.01; *** *p* < 0.001). Our results showed that OR, TY, AR, LE and CA are the most active oils against all tested MDR bacteria and drug-resistant *Candida* species, with inhibition zones average ranging from 18 to 40 mm. The differences in the measured inhibition halos of OR (0.000646), TY (0.000844), CA (0.000532), LE and AR (0.047822), EU (0.057974), LA and SA (0.054462) on *p. aeruginosa* were statistically different from the negative control (without the use of EOs). All tested isolates were sensitive to the EOs of CL and ME (mean inhibition diameter ranging from 12 to 15 mm). Three EOs, LA, SA and EU, displayed low sensitivity against all isolates for which inhibition zones were found to be < 14 mm. The inhibition zones of almost all the EOs were significantly higher than the positive controls represented by cefuroxime (5 ± 2.5 mm) and fluconazole (8 ± 2.5 mm).

### 2.4. MIC, MBC and MFC Values Determination

The ten EOs were evaluated for their inhibitory activity in terms of their minimum inhibitory concentration (MIC) to all isolates. As is evident in Table 4, the tested oils, except those of LA and SA, successfully inhibited the growth of multi-drug-resistant microorganisms. OR, TY and CA displayed very strong activity (MIC 0.025%–0.125% against MDR bacteria, and MIC 0.05% against *Candida* strains). *P. aeruginosa* was the most resistant pathogenic bacteria to all tested EOs.

The MICs, minimum bactericidal concentrations (MBCs), and minimum fungicidal concentrations (MFCs) to all tested strains were almost the same for all the EOs. All the tested EOs, except those of LA, SA and EU, were effective as bactericidal or fungicidal agents (Table 4).

### 2.5. Cytotoxic and DNA-Damaging Effects of EOs

**Figure 3:**
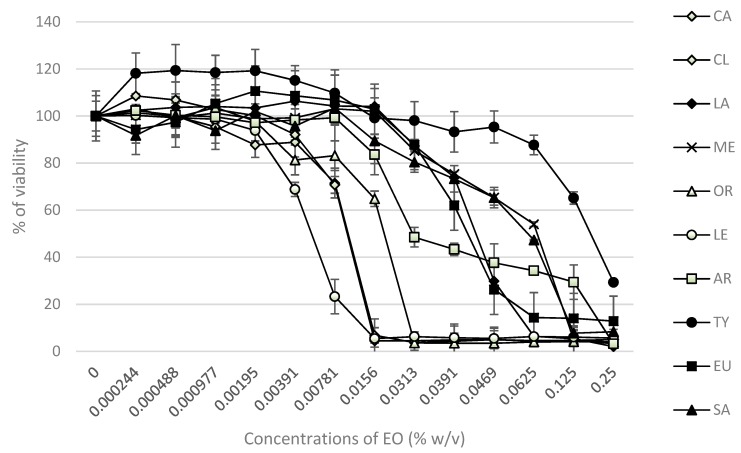
Cytotoxicity/viability of HaCaT cells treated with essential oils (0.00–0.25% *w*/*v*) for 24 h. (CA: cassia; CL: clove; LA: lavender; ME: melaleuca; OR: oregano; LE: lemongrass; AR: arborvitae; TY: thyme; EU: eucalyptus; SA: clary sage).

The cytotoxic effects of 24 h exposure of different concentrations of EOs (0.00–0.25% *w*/*v*) were evaluated in HaCaT cells by the MTT assay. The results are summarized in Figure 3. The IC_50_ values (median inhibitory concentrations that cause approximately 50% cell death) were: 0.0099% for CL and CA; 0.0056% for LE; 0.018% for OR; 0.033% for AR; 0.041% for EU; 0.043% for LA; 0.064% for SA; 0.066% for ME; 0.18% for TY oil.

The genotoxic effects of essential oils occurred at IC~_10–40_. The level of DNA strand breaks induced in HaCaT cells by EOs was determined by the comet assay and was expressed as a % of DNA tail. The studied EOs did not induce DNA damage compared with untreated control HaCaT cells (Table 5). All the studied EOs were highly genotoxic from concentrations of 1.6 × 10^−2^ (arborvitae 8 × 10^−3^%) and above, except for melaleuca (1.28 × 10^−1^).

### 2.6. Total Antioxidant Status Level of Essential Oils

The total antioxidant status (TAS) levels of the EOs and control group are presented in Table 6. Our results show that the 24 h treatment of HaCaT cells with EOs affected the TAS level in a dose-dependent manner. The TAS levels of HaCaT cells in the studied group of EOs were significantly higher than in the negative control. The comparison of the TAS levels of EOs showed that cassia and oregano had the significantly highest values for TAS, at the concentration 2 × 10^−3^% (2.72 ± 0.09; 2.20 ± 0.16). The TAS increased by 403.7% for cassia and 307.4% for oregano in comparison with the negative control. Melaleuca, thyme, clove and clary sage had similar values of TAS at the highest concentrations which were increased by ~170% compared to the negative control. Eucalyptus, arborvitae and lavender increased TAS levels by 127.8%, 87%, and 64.8%, respectively, compared to the negative control.

The lemongrass had the lowest value of TAS compared with the other EOs. When it was applied at the highest concentration (2 × 10^−3^%), the level of TAS increased by 35.2% compared to the negative control.

## 3. Discussion

Recently, there has been a growing interest in the use of natural products in medicine. Research has proven that EOs have remarkable antimicrobial potential and are highly effective against bacteria and *Candida* species [8,15,27]. Several studies have documented that EOs can increase bacterial susceptibility to drugs, even some of the most resistant strains [3,15,28]. EOs are generally found in plants as mixtures of various active components, especially monoterpenes (phenols and sesquiterpenes), and their antimicrobial efficacy depends on its chemical composition [29,30]. In our study, we selected EOs which are well-known for their high content of phenols (OR, TY, CL), Eos with aldehydes content (CA, LE), terpene alcohols (ME, LA), oxides (EU), esters (SA), and a phenol-free EO (AR). The multi-drug-resistant (MDR) bacteria and *Candida* species used for this study were chosen based on previous findings about the ability of some EOs to inhibit certain human pathogens [11,19,31,32]. The measured inhibition halos of OR, TY, AR, LE, CA, CL, ME, EU, LA, and SA indicated that all of the EOs are effective against MDR bacteria and drug-resistant *Candida* species, with inhibition zones, on average, ranging from 18 to 40 mm for 10 µL of EOs. The OR, TY and CA used in this study were even more effective than the antibiotic cefuroxime and fluconazole. Our results are in accordance with a previous study showing that the inhibitory halos produced by the EOs of OR, TY, and CL were larger than those produced by ciprofloxacin [33] and cefuroxime [11]. Grullon et al. [34] tested EOs from cassia, cinnamon bark, cinnamaldehyde, and methylglyoxal using the disc diffusion method. The study showed that cinnamaldehyde and methylglyoxal were as effective or better in inhibiting the growth of *P. aeruginosa* compared to standard aminoglycoside antibiotics.

Sharifzadeh et al. [32] determined the antifungal activity of the EOs of *Origanum vulgare, Myrtus communis, Zingiber officinale roscoe, Matricaria chamomilla* and *Trachyspermum ammi*, against both fluconazole (FLU)-resistant and FLU-susceptible *C. albicans* strains isolated from patients with oropharyngeal candidiasis. The main finding was that the susceptibility of FLU-resistant *C. albicans* to EOs was higher than those of the FLU-susceptible yeasts. Similarly, Soares et al. [35] confirmed the antifungal activity of EOs from OR, TY and cinnamon against FLU-susceptible and FLU-resistant *Candida glabrata.* In yeast, various mechanisms of cell destruction have been observed in EOs. These mechanisms mainly depend on the chemical composition of EOs. The mechanism of action class of compounds (phenols) presupposes the disruption of cell membranes and destruction of yeast mitochondria [36].

The low antimicrobial activity of the LA, SA and EU EOs may be due to the relatively low phenol content of these EOs: their main components are alcohols [37], esters [38] and oxides. This is in accordance with a known study on the antimicrobial efficiency of the Eos from *Salvia officinalis*, which reported a very low antibacterial activity for 1,8-cineole against *S. aureus, B. subtilis*, and *E. coli* [39]. Similarly, Puškárová et al. [16] confirmed that LA and SA were both less active against pathogenic bacteria (*E. coli, S. typhimurium, Y. enterocolitica, S. aureus, L. monocytogenes, E. faecalis*) and environmental bacteria (*B. cereus, A. protophormiae, P. fragi*), with inhibition zones ranging from 8 to 14 mm. The disc diffusion assay is limited by the hydrophobic nature of most EOs, which prevents their uniform diffusion through the agar medium; therefore, most researchers prefer liquid medium methods [40]. The EOs of OR, TY and CA exhibited strong antimicrobial activity against all the microorganisms in liquid medium, as has been previously described [13,41]. It is important to mention that EOs are more active against Gram-positive than against Gram-negative bacteria [13,15,19], presumably due to differences in cell wall composition [15,42,43]. Preuss et al. [44] found that OR is lethal to *E. coli* and *Klebsiella pneumoniae*. Essential oils from OR, TY and CL were effective against the Gram-negative bacteria *E. coli* O157:H7, *Y. enterocolitica* O9, *Proteus* spp., and *K. pneumoniae* [45]. Our results have also shown that tested Gram-negative bacteria (*K. pneumoniae, P. aeruginosa, P. vulgaris, C. koseri*) are sensitive to OR, TY, CA, CL, AR, and ME. Thymol, eugenol, and carvacrol have an antimicrobial effect against a broad spectrum of bacteria, namely *Escherichia coli, Bacillus cereus, Listeria monocytogenes, Salmonella enterica, Clostridium jejuni, Lactobacillus sake, Staphylococcus aureus,* and *Helicobacter pyroli* [15,22,23,26]. García-Salinas et al. [46] found that the carvacrol, cinnamaldehyde, and thymol molecules contained in EOs could be used against *E. coli-* and *S. aureus*-mediated infections, without a potential induction of bactericidal resistance and with lower cell toxicity. Other families of EO compounds also show antimicrobial properties: certain alcohols, aldehydes, ketones, monoterpene (geraniol, linalol, menthol, terpineol, thujanol, thujone, camphor etc.), phenylpropanes (cinnamaldehyde), and monoterpenes (-terpinene, p-cymene) [15]. 

Generally, the cytotoxicity of EOs mainly correlates to the presence of phenols, alcohols, and monoterpene aldehydes [15,47,48]. The cytotoxic effect of EOs was investigated in vitro on human normal keratinocyte cell lines using an MTT assay. The results of the MTT assay showed that the 24 h treatment of cells with EOs affected cell viability in a dose-dependent manner; IC_50_ values declined in order TY > ME > SA > LA > EU > AR > OR > LE > CA = CL. These results for the IC_50_ values of EOs are in good correlation with other studies where HeLa and human normal colon cell lines were used [49,50].

According to our previous data using human embryo lung cells (HEL 12469), the cytotoxic effect of the studied EOs was detected in similar concentrations (IC_50_). The IC_50_ values in HEL 12469 cells declined in the order SA > LA > AR = TY > CL > OR, which is very similar to our current results [16].

Adukwu et al. [47] have investigated the effect of LE and its major component, citral, on the viability of human dermal fibroblasts. They found that the components exhibited higher cytotoxic effects than LE alone. This can be explained by the fact that, in addition to citral, other components are present at different concentrations, which can act antagonistically and reduce the toxic profile of the oil. Another complication of EOs and their components is their hydrophobic nature. Such problems can be solved, e.g., by binding various substrates to individual components that would reduce the toxicity and hydrophobic nature of EOs.

Our results show that the EO of lemongrass, for instance, is toxic to HaCat cells at concentrations of IC_50_ 0.0056% or higher, while the EOs of cassia, clove and oregano are toxic above 0.01% and other oils are well tolerated up to concentrations of ~0.05%. The EO of thyme was the least toxic for HaCaT cell lines (IC_50_ 0.18%). These results are in very good correlation with a previous study from Spagnoletti et al. [51], where the EOs of oregano, thyme and rosemary, containing carvacrol and thymol, were cytotoxic only at high concentrations (IC_50_ > 130 μg/mL) in HaCaT cell lines. The same results were obtained by LLana-Ruiz-Cabello et al. [52] and Slamenova et al. [53], where they studied the effects of thymol and carvacrol on the digestive tract and liver using CaCo-2 and HepG2 models.

The level of DNA strand breaks induced in HaCaT cells by the studied EOs was determined by the comet assay. The treatment with EOs did not induce any significant increase in DNA strand breaks compared to the untreated control cells. The select concentrations of EOs for comet assay were not cytotoxic. These results are in very good correlation with our previous study, where Eos, similarly, did not induce DNA damage in the HEL 12469 cells [16]. A comparable effect was also shown using the plant extracts of *Salvia officinali* and *Thymus vulgaris* using HepG2 and primary rat hepatocytes [54,55].

Our goal was to determine the EO concentration level that is able to inhibit the growth of microorganisms and, at the same time, lacks cytotoxicity in HaCaT cells. Selected bacterial strains are Gram-negative, due to the outer membrane being more resistant to EOs and antibiotics. Based on our results, we can conclude that EOs are able to destroy multiresistant bacterial strains in a dose-dependent manner. On the other hand, the toxicity of individual EOs raises some concerns. Thymol showed the highest effectivity and it is the only EO where we determined the concentration that had an antimicrobial effect and simultaneously did not have cytotoxic effect in HaCaT.

Oxidative stress is characterized by an abnormal quantity of reactive oxygen species in the body. Antioxidants are compounds that, with different mechanisms, dampen or counteract oxidative stress, either by reducing the cause or the consequences of oxidative stress. Antioxidant enzymes such as superoxide dismutase, catalase, and glutathione peroxidase serve as a primary line of defense in destroying free radicals [56]. Polyphenols, which contain two or more phenol groups, are ubiquitous in plant foods. In general, their effectiveness in protecting against oxidative stress depends on their reactivity towards free radicals. Flavonoids, the largest group of polyphenols, contain strong antioxidants such as quercetin or catechin, which can interact with intracellular antioxidative species such as glutathion peroxidase, and may enhance their antioxidative activities [57,58]. Our results showed that the 24 h treatment of HaCaT cells with EOs affected TAS levels in a dose-dependent manner. These results are in accordance with previous studies [59,60,61], where essential oils increased the antioxidant effects in in vitro and in vivo systems (HepG2, IEC-6, hepatocytes of rat and mice). The results of Placha et al. [62] demonstrated that lower concentrations of EOs improve the health status of animals. Total antioxidant status in plasma significantly increased in a group of birds with their diet supplemented with an EO (sage oil).

## 4. Materials and Methods

### 4.1. Essential Oils

The study was performed employing the following ten EOs: oregano (OR) (*Origanum vulgare* L.), thyme (TY) (*Thymus vulgaris* L.), clove (CL) (*Eugenia caryophyllata* L.), arborvitae (AR) (*Thuja plicata* Donn.), cassia (CA) (*Cinnamomum cassia* NO.), lemongrass (LE) (*C. flexuosusand* DC.) melaleuca (ME) (*Melaleuca alternifolia* Cheel.), eucalyptus (EU) (*Eucalyptus radiata* Sieber ex DC.), lavender (LA) (*Lavandula angustifolia* Mill.), and clary sage (SA) (*Salvia sclarea* L.).

All test EOs were acquired from the producer doTERRA International (Pleasant Grove, UT, USA). For screening, 1% dilution (*w*/*v*) in dimethylsulfoxide (DMSO, Sigma–Aldrich Co., Saint-Louis, MO, USA) was prepared (i.e., 10 mg EO, diluted to 1 mL with DMSO).

### 4.2. Gas Chromatography–Mass Spectrometry Analysis–Chemical composition

The volatile compounds of essential oils were analyzed on a 6890N gas chromatograph (Agilent Technologies, Santa Clara, CA, USA) equipped with a non-polar (5%-Phenyl)-methylpolysiloxane DB-5 column (length 30 m, inner diameter 250 µm, film thickness 0.5 µm) (Agilent Technologies) and coupled to a 5973 mass spectrometric detector (Agilent Technologies). A temperature programme of 40 °C for 1 min, 5 °C/min and 200 °C for 1 min was used. The split ratio was 30:1. The average velocity of helium carrier gas was 45 cm/s with constant flow. An ionization voltage (EI) of 70 eV was used. The identification of volatile compounds was done by comparison of mass spectra with NIST 14 MS library (National Institute Standards and Technology, Gaithersburg, MD, USA).

### 4.3. Antimicrobial Activity

Each EO was tested against 4 MDR bacterial strains: (*Klebsiella pneumoniae* KMB 522, *Pseudomonas aeruginosa* KMB 527, *Proteus vulgaris* KMB 525, and *Citrobacter koseri* KMB 526) and 3 clinical DR *Candida* species were used. One was a fluconazole-resistant *Candida albicans* strain, the second was a fluconazole- and voriconazole- resistant *Candida parapsilosis* strain, and the third was a voriconazole-resistant and fluconazole-sensitive *Candida parapsilosis* strain. All the isolates were obtained from patients with skin infections (from Comenius University Faculty of Medicine in Bratislava, Slovakia), and their resistance against 15 antibiotics and 2 antimycotics was verified using Vitek 2 (bioMérieux CZ): cefuroxime (CEF), cefotaxime (CTX), ampicillin (AMP), ampicillin + sulbactam (AMS), sulbactam (SUB), tetracycline (TET), ceftazidime (CAZ), tigecykline (TIG), cefepime (CPM), tobramycin (TOB), clotrimazole (CLM), ciprofloxacin (CIP), amikacin (AMI), colistin (COL) and gentamycin (GEN), and fluconazole (FLU) and voriconazole (VOR) (Table 1 and Table 2). The bacterial and yeast strains were stored at −80 °C in a glycerol broth.

### 4.4. EOs Agar Disc Diffusion Assay

The antibacterial and antifungal activities of each EO were determined by using the paper disc diffusion method to screen the efficacy of essential oils following the previously described procedure. An amount of 10 μL of each EO, at a concentration 100%, was applied on a sterile paper disc (6 mm Ø Whatman No.1) aseptically placed on the inoculated plates [11]. The average of inhibition zones was evaluated classifying the EOs as follows: not sensitive for a diameter smaller than 8 mm; sensitive for a diameter of 9–14 mm; very sensitive for a diameter of 15–19 mm; extremely sensitive for a diameter larger than 20 mm [35]. Cefuroxime (30 μg/disc; Sigma–Aldrich., Saint-Louis, MO, USA) and fluconazole (20 μg/disc; Sigma–Aldrich, Saint-Louis, MO, USA) were used as positive controls for bacterial and fungal inhibition. After 24 h of incubation at 37 °C, the inhibition zones were measured in millimeters, including the diameter of the disc. Regarding cefuroxime and fluconazole, for areas smaller than 7 mm and 17 mm in diameter the inhibitory effects were classified as not sensitive, respectively. The results were classified according Eucast (http://www.eucast.org). All experiments were conducted in triplicate.

### 4.5. Determining Minimal Inhibitory Concentrations (MIC) of EOs

The MIC values of the ten EOs against four MDR bacteria and three drug-resistant *Candida* strains were estimated using the micro-well dilution method described previously [11] with modifications. With sterile, round-bottom 96-well plates, duplicate two-fold serial dilutions of each EO (100 μL/well) were prepared in the suitable broth (Mueller–Hinton (MHB) or Sabouraud Dextrose broth (SDB) containing 5% (*v*/*v*) DMSO to establish a range of concentrations (0.05%, 0.10%, 0.50%, 1.0%, 2.5%, 5.0%, and 10% (*w*/*v*) of EOs. An amount of 100 μL (1 × 10^6^ CFU/mL) of the bacterial or yeast suspension, which was previously prepared in the proper broth, was then added in each well. After that, the 96-well plates were incubated at 37 °C for 24 h for the bacteria, and 48 h for the *Candida* strains, under aerobic conditions. The MIC of each oil was defined as the lowest EO concentration exhibiting no detectable bacterial or fungal growth.

For the determination of the MBC and MFC (minimal bactericidal and minimal fungicidal concentration), part of the liquid (20 μL) from each well that exhibited no growth was taken and incubated on the proper agar plates at 37 °C for a further 24 h for bacteria, and 48 h for *Candida* species. The lowest concentration of EO that revealed no visible bacterial or fungal growth was considered as the MBC or MFC. The lowest concentration that revealed no visible bacterial or fungal growth (at which no growth occurred on the MHA and SDA plates) was considered as MBC or MFC.

### 4.6. Cell Culture

The human keratinocyte cell line HaCaT (T0020001) was purchased from AddexBio (San Diego, USA). The cells (HaCaT) were cultivated in Dulbecco’s Modified Eagle Medium (DMEM) supplemented with 10% fetal calf serum (FCS) and antibiotics (penicillin 100 U/mL; streptomycin 100 μg/mL). The cells were cultured in a humidified atmosphere of 5% CO_2_ at 37 °C. The media and chemicals used for cell cultivation were purchased from Gibco BRL (Paisley, UK).

### 4.7. Determination of Cytotoxicity

The metabolic activity of EO was determined using the 3-(4,5-dimethyl-thiazoyl)-2,5-diphenyltetrazolium bromide (MTT) method. Briefly, 2 × 10^4^ cells were seeded in 96-well plates and cultured in complete DMEM medium. The studied EOs (0.00%–0.25% *w*/*v*) were then added, and the cells were incubated at 37 °C in a 5% CO_2_ atmosphere for 24 h. At the indicated time point, the samples were washed with phosphate buffered saline (PBS), followed by incubation with 1 mg/mL of MTT for 4 h. Then, the MTT was removed and the formazan crystals were dissolved with dimethyl sulfoxide for 30 min. As a positive control, hydrogen peroxide (300 μmol/L, Saint-Louis, MO, USA) was used. Absorbance at a wavelength of 540 nm was measured using an xMark™ Microplate Spectrophotometer (Bio-Rad Laboratories, Inc., Hercules, CA, USA) and background absorbance at 690 nm was subtracted.

### 4.8. Determination of Genotoxicity

Exponentially growing cells were pre-incubated in the presence of EOs (6.25 × 10^−5^–8 × 10^−3^% *w*/*v*), melaleuca (5 × 10^−4^–6.4 × 10^−2^% *w*/*v*), or without EOs (control) for 24 h. Then the cells were washed, trypsinized, re-suspended in a fresh culture medium and used for testing of the level of DNA lesions using the comet assay. The procedure was used with minor modifications suggested by [63]. The slides were examined with a Zeiss Imager.Z2 fluorescence microscope using the computerized image analysis (Metafer 3.6, Meta Systems GmbH, Altlussheim, Germany). The percentage of DNA in the tail (% of tail DNA) was used as a parameter for the measurement of DNA damage (DNA strand breaks). One hundred comets were scored per each sample in one electrophoresis run.

### 4.9. Determination of Total Antioxidant Status (TAS)

The TAS was determined by a chromogenic method (Randox Laboratories, UK) with minor adaptations. This methodology was based on the capacity to inhibit the formation of the ABTS^+^ radical cation (2,2′-azino-di-[3-etylbenzotiazolin sulphonate]). Absorbance at a wavelength of 600 nm was measured using a spectrophotometer xMark™ Microplate Spectrophotometer (Bio-Rad Laboratories, Inc., Hercules, CA, USA). As the positive control, ascorbic acid (10 µmol/L, Sigma–Aldrich) was used. Results are expressed as μmol of TAS per gram of protein (μmol/g prot). The protein concentrations were determined using the Bradford method [64].

### 4.10. Statistical Analysis

The results represent a mean from 3 to 5 experiments ± standard deviation (SD). The differences between the defined groups were tested for statistical significance using Student’s *t*-test (* *p* < 0.05; ** *p* < 0.01; *** *p* < 0.001).

## 5. Conclusions

The essential oils exhibited antimicrobial and antioxidant activity, and may be used as a potential source of safe and potent natural antimicrobial and antioxidant agents in the pharmaceutical and food industries. Their low cytotoxicity and antioxidant efficacy at non-genotoxic concentrations may facilitate their use in the treatment of skin and other infections where the local application of antimicrobial agents is possible.

## Figures and Tables

**Table 1:** Gas chromatography–mass spectrometry analysis of ten essential oils.

**Oregano**	*Origanum vulgare*	Carvacrol (76.73%), Thymol (11.34%), *p*-cymene (4.67%)
**Thyme**	*Thymus vulgaris*	Thymol (53.26%), Carvacrol (6.27%), *p*-cymene (16.8%), γ-Terpinene (6.48%), Linalool (4.05%), β-Caryophyllene (1.82%)
**Clove**	*Eugenia caryophyllata*	Eugenol (77.83%), Eugenyl acetate (14.22%), β-Caryophyllene (5.07%)
**Arborvitae**	*Thuja plicata*	Methyl thujate (55.96%), Methyl myrtenate (6.21%), Terpinen-4-ol (2.97%), α-Terpineol (2.06%)
**Cassia**	*Cinnamomum cassia*	*trans*-Cinnamaldehyde (87.85%), *o*-methoxycinnamaldehyde (5.36%)
**Lemongrass**	*Cymbopogon flexuosus*	Geranial (45.72%), Neral (34.46%), Geraniol (5.99%), Geranyl acetate (3.83%)
**Melaleuca**	*Melaleuca alternifolia*	Terpinen-4-ol (44.48%), γ-Terpinene (16.84%), α-Terpinene (6.40%)
**Eucalyptus**	*Eucalyptus radiata*	Eucalyptol (73.82%), α-Terpineol (9.88%)
**Clary sage**	*Salvia sclarea*	Linalyl acetate (53.65%), Linalool (22.32%), α-Terpineol (5.93%), Geranyl acetate (4.32%), Neryl acetate (2.37%)
**Lavender**	*Lavandula angustifolia*	Linalyl acetate (29.15%), Linalool (30.07%), Terpinen-4-ol (4.66%), Lavandulyl acetate (5.56%), β-Caryophyllene (4.16%), cis-β-Ocimene (3.93%)

**Table 2:** Susceptibility to antibiotics of multi-drug-resistant bacteria.

Bacteria	CEF	CTX	CAZ	CPM	SUB	AMP	AMS	TIG	TET	CLM	CIP	COL	GEN	TOB	AMI
*P. aeruginosa* KMB527	R	R	S	R	R	S	R	S	R	R	R	S	R	R	R
*P. vulgaris* KMB525	R	R	S	R	S	R	R	R	R	R	R	R	R	R	S
*K. pneumoniae* KMB522	R	R	R	R	S	R	R	S	S	R	R	S	R	R	S
C. *koseri* KMB526	R	R	S	R	S	R	R	R	R	R	R	R	R	R	S

CEF: cefuroxime; CTX: cefotaxime; CAZ: ceftazidime; CPM cefepime; SUB: sulbactam; AMP: ampicillin; AMS: ampicillin + sulbactam; TIG: tigecykline; TET: tetracycline; CLM: clotrimazole; CIP: ciprofloxacin; COL: colistin; GEN: gentamicin; TOB: tobramycin; AMI: amikacin; R: resistant; S: susceptible.

**Table 3:** Susceptibility to antimycotics of drug-resistant yeasts.

*Candida* Strains	FLU	VOR
*C. albicans* Nr. 2	R	S
*C. parapsilosis* Nr. 8	S	R
*C. parapsilosis* Nr. 52	R	R

FLU fluconazole; VOR voriconazole; R: resistant; S: susceptible.

**Table 4:** Minimum inhibitory concentrations (MIC; % *w*/*v*), minimum bactericidal concentrations (MBC; % *w*/*v*), and minimum fungicidal concentrations (MFC; % *w*/*v*) of several EOs against tested microorganisms.

Microorganisms		Activity	OR	TY	CL	AR	CA	LE	ME	EU	LA	SA
**Bacteria**	*P. aeruginosa* KMB527	MIC	0.125	0.125	0.5	0.25	0.125	0.25	0.5	2.5	-	-
		MBC	0.125	0.125	0.5	0.25	0.125	0.25	0.5	-	-	-
	*P. vulgaris* KMB525	MIC	0.05	0.05	0.125	0.125	0.025	0.25	0.125	1.25	0.5	0.5
		MBC	0.05	0.05	0.125	0.125	0.025	0.25	0.125	1.25	0.5	0.5
	*K. pneumoniae* KMB522	MIC	0.05	0.05	0.125	0.125	0.125	0.25	0.125	1.25	0.5	0.5
		MBC	0.05	0.05	0.125	0.125	0.125	0.25	0.125	1.25	0.5	0.5
	*C. koseri* KMB526	MIC	0.025	0.025	0.125	0.05	0.05	0.25	0.025	1.25	0.5	0.5
		MBC	0.025	0.025	0.125	0.05	0.05	0.25	0.025	1.25	0.5	0.5
**Yeasts**	*C. albicans* Nr. 2	MIC	0.05	0.05	0.5	0.05	0.125	0.125	0.125	2.5	-	-
		MFC	0.05	0.05	0.5	0.05	0.125	0.125	0.125	-	-	-
	*C. parapsilosis* Nr. 8	MIC	0.05	0.05	0.5	0.05	0.125	0.125	0.125	2.5	-	-
		MFC	0.05	0.05	0.5	0.05	0.125	0.125	0.125	-	-	-
	*C. parapsilosis* Nr. 52	MIC	0.05	0.05	0.5	0.05	0.125	0.125	0.125	2.5	-	-
		MFC	0.05	0.05	0.5	0.05	0.125	0.125	0.125	-	-	-

OR: oregano; TY: thyme; CL: clove; AR: arborvitae; CA: cassia; LE: lemongrass; ME: melaleuca; EU: eucalyptus; LA: lavender; SA: clary sage; -: no growth inhibition.

**Table 5:** The levels of DNA single strand breaks (% of tail DNA) in HaCaT cells after the exposure to essential oils for 24 h. Data represent means ± SD of three independent experiments.

EOs	Concentrations of EOs											
	0	6.25 × 10^−5^	1.25 × 10^−4^	2.5 × 10^−4^	5 × 10^−4^	1 × 10^−3^	2 × 10^−3^	4 × 10^−3^	8 × 10^−3^	1.6 × 10^−2^	3.2 × 10^−2^	6.4 × 10^−2^
**Clary sage**	7.62 ± 0.80	8.92 ± 0.10	9.21 ± 0.99	8.98 ± 0.90	8.68 ± 0.11	9.59 ± 0.26	7.88 ± 0.30	6.93 ± 0.90	7.71 ± 0.99	ND	ND	ND
**Clove**	8.65 ± 0.27	9.71 ± 1.09	8.91 ± 1.16	7.18 ± 0.75	8.03 ± 1.42	8.50 ± 1.31	7.09 ± 1.91	8.44 ± 0.63	7.61 ± 0.99	ND	ND	ND
**Oregano**	7.61 ± 1.15	9.29 ± 0.88	7.71 ± 1.45	8.82 ± 0.86	9.73 ± 0.98	9.93 ± 1.54	7.64 ± 1.47	8.27 ± 0.49	8.69 ± 0.53	ND	ND	ND
**Lemongrass**	9.86 ± 1.22	11.21 ± 1.46	9.95 ± 1.61	11.93 ± 1.16	10.28 ± 1.19	11.62 ± 1.46	10.74 ± 1.73	10.92 ± 1.15	9.63 ± 1.12	ND	ND	ND
**Melaleuca**	8.34 ± 0.58	8.29 ± 0.88	7.82 ± 0.86	7.50 ± 1.31	7.55 ± 1.04	7.19 ± 0.96	8.17 ± 1.22	8.18 ± 0.96	7.49 ± 0.66	7.56 ± 0.37	7.20 ± 0.42	7.61 ± 0.79
**Arborvitae**	8.29 ± 0.67	9.31 ± 1.92	9.11 ± 2.19	10.37 ± 0.89	9.07 ± 1.57	8.18 ± 1.22	10.06 ± 1.60	8.89 ± 0.66	ND	ND	ND	ND
**Cassia**	10.12 ± 0.66	9.33 ± 1.06	10.04 ± 1.27	8.73 ± 0.79	7.89 ± 0.78	9.86 ± 1.34	10.57 ± 0.90	8.89 ± 0.52	10.58 ± 1.11	ND	ND	ND
**Lavender**	9.24 ± 0.60	8.29 ± 1.36	8.61 ± 0.82	9.02 ± 1.32	7.69 ± 1.20	9.25 ± 1.00	8.27 ± 1.30	8.76 ± 0.81	9.53 ± 1.43	ND	ND	ND
**Thyme**	8.27 ± 1.32	9.29 ± 0.66	9.28 ± 1.04	9.02 ± 0.33	9.02 ± 1.18	9.82 ± 0.29	8.60 ± 1.37	10.09 ± 0.60	9.53 ± 1.43	ND	ND	ND
**Eucalyptus**	8.24 ± 0.54	7.59 ± 1.20	9.32 ± 0.32	8.58 ± 1.25	8.93 ± 1.58	9.69 ± 1.19	8.42 ± 1.70	8.40 ± 1.04	8.04 ± 0.68	ND	ND	ND

ND: (not detectable); positive control - hydrogen peroxide (300 μmol/L), 50.94 ± 1.76.

**Table 6:** Total antioxidant status (TAS) in HaCaT cells exposed to essential oils for 24 h.

Essential Oil	Dose (% *w*/*v*)	TAS (mmol/prot)
**Control (−)**	-	0.54 ± 0.04
**Control (+)**	-	4.32 ± 0.08
**Lemongrass**	8 × 10^−3^	0.56 ± 0.07
	4 × 10^−3^	0.71 ± 0.07 **
	2 × 10^−3^	0.73 ± 0.03 **
**Clove**	8 × 10^−3^	0.74 ± 0.12**
	4 × 10^−3^	1.03 ± 0.08 **
	2 × 10^−3^	1.45 ± 0.06 **
**Oregano**	8 × 10^−3^	1.53 ± 0.08 **
	4 × 10^−3^	2.07 ± 0.13 ***
	2 × 10^−3^	2.20 ± 0.16 ***
**Melaleuca**	6.4 × 10^−2^	0.78 ± 0.12 **
	3.2 × 10^−2^	1.19 ± 0.06 ***
	1.6 × 10^−2^	1.51 ± 0.28 **
**Cassia**	8 × 10^−3^	1.47 ± 0.11 **
	4 × 10^−3^	2.01 ± 0.14 ***
	2 × 10^−3^	2.72 ± 0.09 ***
**Arborvitae**	4 × 10^−3^	0.42 ± 0.08
	2 × 10^−3^	0.91 ± 0.13 **
	1 × 10^−3^	1.01 ± 0.08 **
**Lavender**	8 × 10^−3^	0.61 ± 0.05 *
	4 × 10^−3^	0.65 ± 0.09 *
	2 × 10^−3^	0.89 ± 0.07 **
**Thyme**	8 × 10^−3^	0.77 ± 0.11 *
	4 × 10^−3^	1.13 ± 0.09 ***
	2 × 10^−3^	1.47 ± 0.12 **
**Clary sage**	8 × 10^−3^	0.75 ± 0.04 **
	4 × 10^−3^	1.31 ± 0.11 **
	2 × 10^−3^	1.41 ± 0.10 **
**Eucalyptus**	8 × 10^−3^	0.77 ± 0.08 **
	4 × 10^−3^	0.93 ± 0.05 **
	2 × 10^−3^	1.23 ± 0.05 ***

Data represent mean ± SD of three independent experiments. * *p* < 0.05; ** *p* < 0.01; *** *p* < 0.001 indicate significant differences compared to untreated control cells.

## Supplementary Materials

The following are available online. Retention indexes of several EO compounds (Table S1) and the spectra of 10 EOs analyzed by gas-chromatography-mass spectrometry (Figure S1).
