# Supplementary material for: The Effect of Ten Essential Oils on Several Cutaneous Drug-Resistant Microorganisms and Their Cyto/Genotoxic and Antioxidant Properties
Source: Molecules. 2019 Dec 13;24(24):4570. doi: 10.3390/molecules24244570 (PMC6943746; doi:10.3390/molecules24244570)

# Supplementary Material

# The effect of ten essential oils on several cutaneous drug resistant microorganisms and their cyto/genotoxic and antioxidant properties

**Katarína Kozics[1], Mária Bučková[2], Andrea Puškárová[2], Viktória Kalászová[3], Terézia Cabicarová[4] and Domenico Pangallo[2]***

[1] Cancer Research Institute BMC, Slovak Academy of Sciences, Dúbravska cesta 9, 845 05 Bratislava, Slovakia; katarina.kozics@savba.sk

[2] Institute of Molecular Biology, Slovak Academy of Sciences, Dúbravská cesta 21, 84551 Bratislava, Slovakia; maria.buckova@savba.sk, andrea.puskarova@savba.sk

[3] Department of Genetics, Faculty of Natural Sciences, Comenius, University, Mlynská dolina, 842 15 Bratislava, Slovakia, viktoria.kalaszova@gmail.com

[4] Food Research Institute, National Agricultural and Food Centre, Priemyselná 4, 824 75 Bratislava, Slovakia; tereza.cabicarova@nppc.sk

* Correspondence: domenico.pangallo@savba.sk; Tel.: (+421259307439); fax: +421 2 5930 7416

**Table S1.** Retention indexes of several compounds present in tested essential oils

| **Compound name** | **Retention index (RI)** |
|---|---|
| Carvacrol | 1303,4 |
| Thymol | 1294,2 |
| *para*-Cymene | 1024,3 |
| $\alpha$-Terpinene | 1016,4 |
| $\gamma$-Terpinene | 1058,8 |
| Linalool | 1100,9 |
| Caryophyllene /β-Caryophyllene/ | 1423,8 |
| Eugenol | 1360,1 |
| Eugenyl acetate | 1530,6 |
| Methyl thujate | 1325,7 |
| Methyl myrtenate | 1299,7 |
| Terpinen-4-ol | 1179,1 |
| $\alpha$-Terpineol | 1192,3 |
| trans-Cinnamaldehyde | 1272,5 |
| o-Methoxycinnamaldehyde | 1534,8 |
| Geranial | 1272,6 |
| Neral | 1243,1 |
| Geraniol | 1256,6 |
| Geranyl acetate | 1385,7 |
| Eucalyptol | 1031,0 |
| Linalyl acetate | 1257,6 |
| Neryl acetate | 1366,5 |
| Lavandulyl acetate | 1292,5 |
| cis-β-Ocimene | 1038,4 |

RI - Retention index relative to n-alkanes on column Ultra 2 (25m x 0.2 mm x 0.33μm). Temperature program 40 °C, 3 °C/min, 250 °C. Pressure 85 kPa, constant pressure.

**Figure S1.** The spectra of gas-chromatography-mass spectrometry analysis of 10 essential oils.

**Oregano**

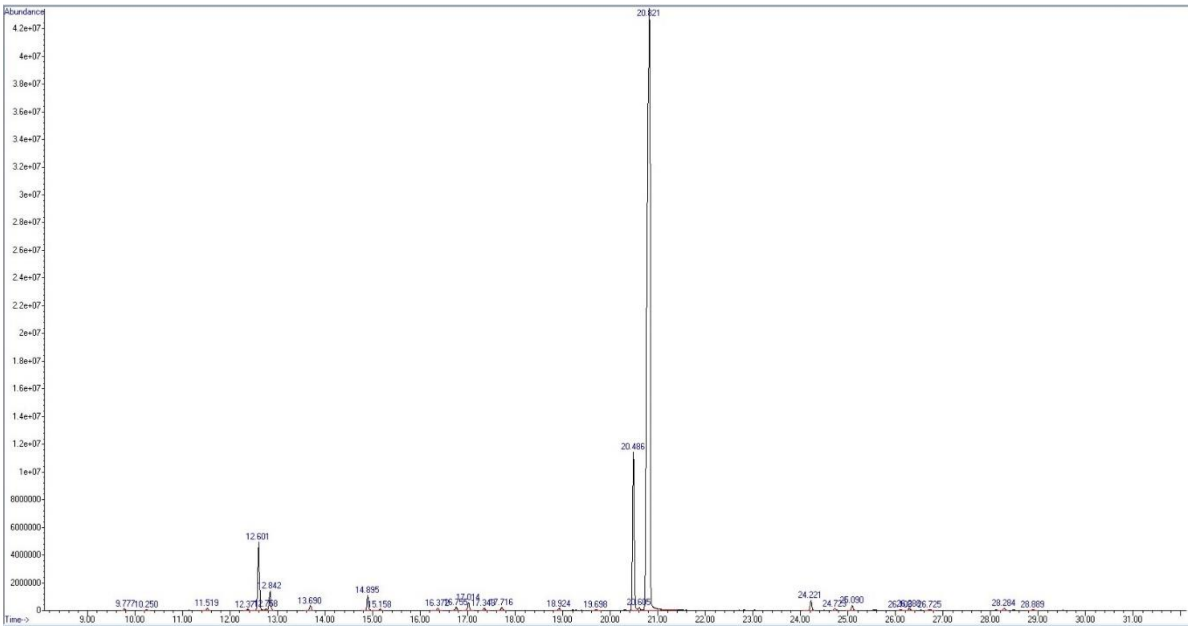


**Thyme**

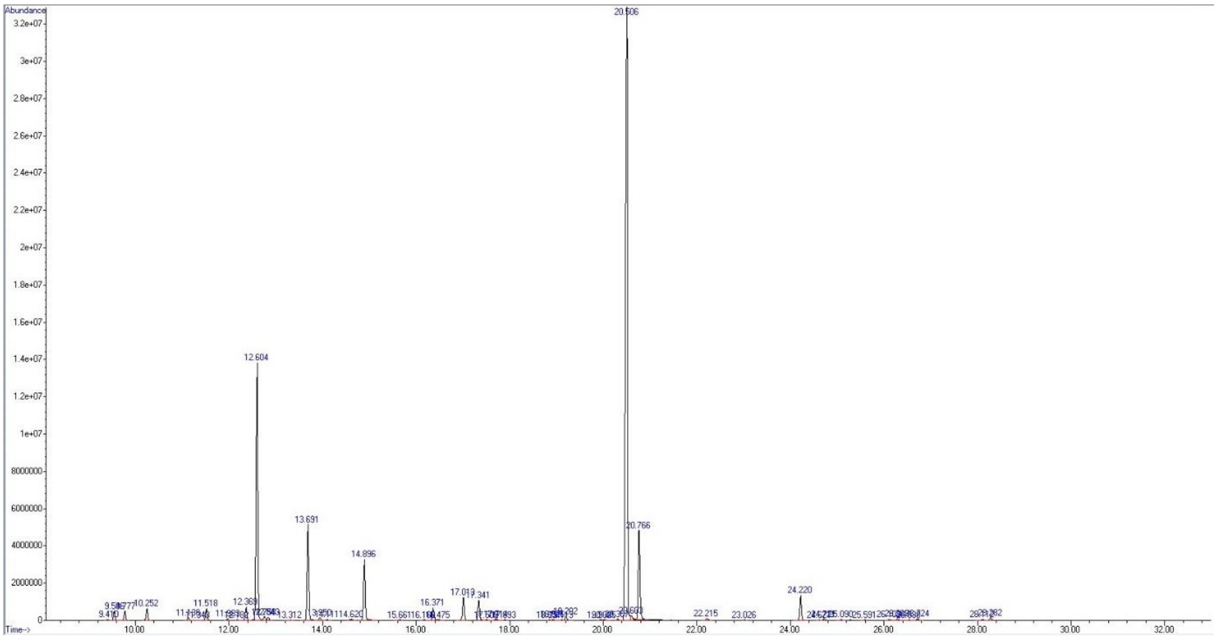

**Clove**

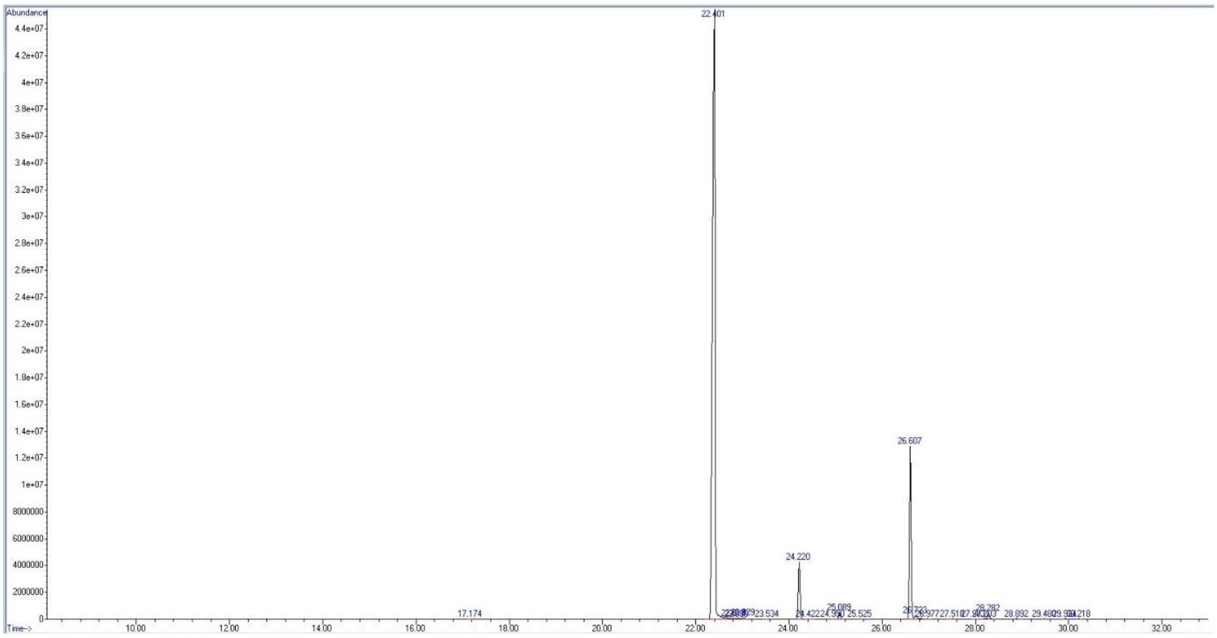


**Arborvitae**

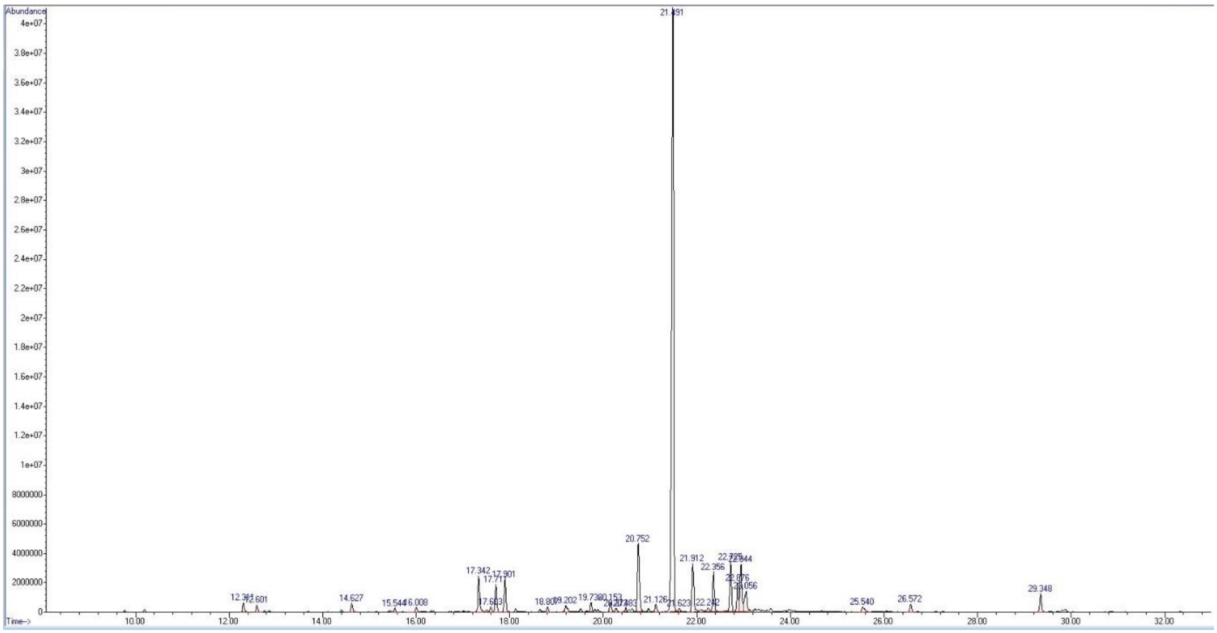

**Cassia**

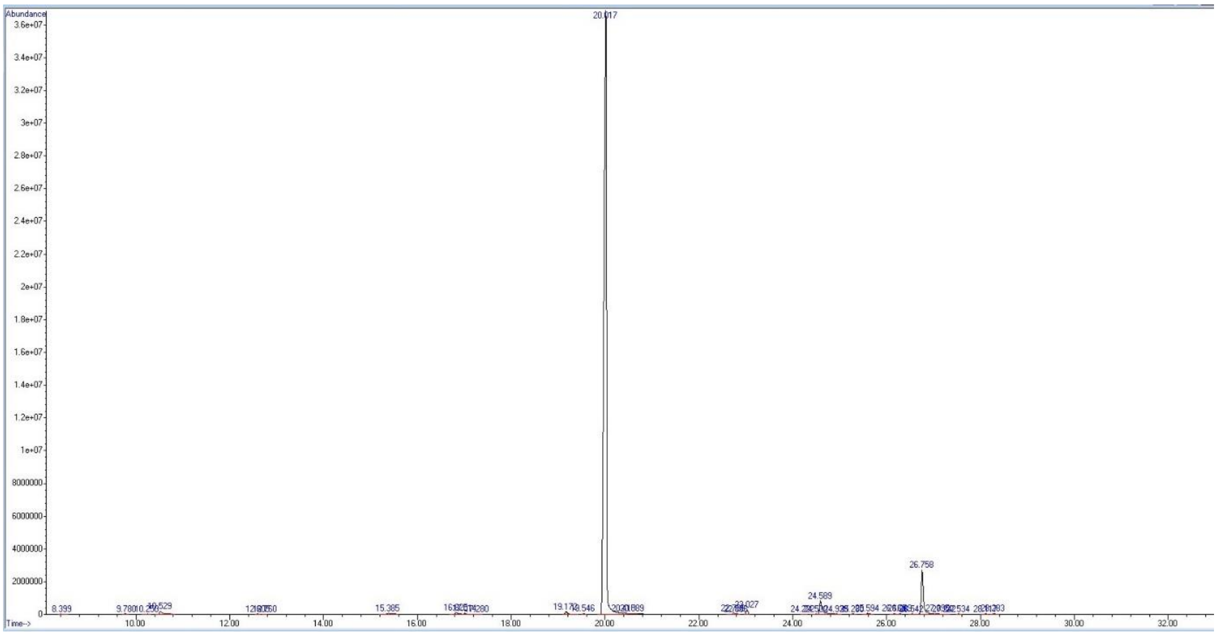


**Lemongrass**

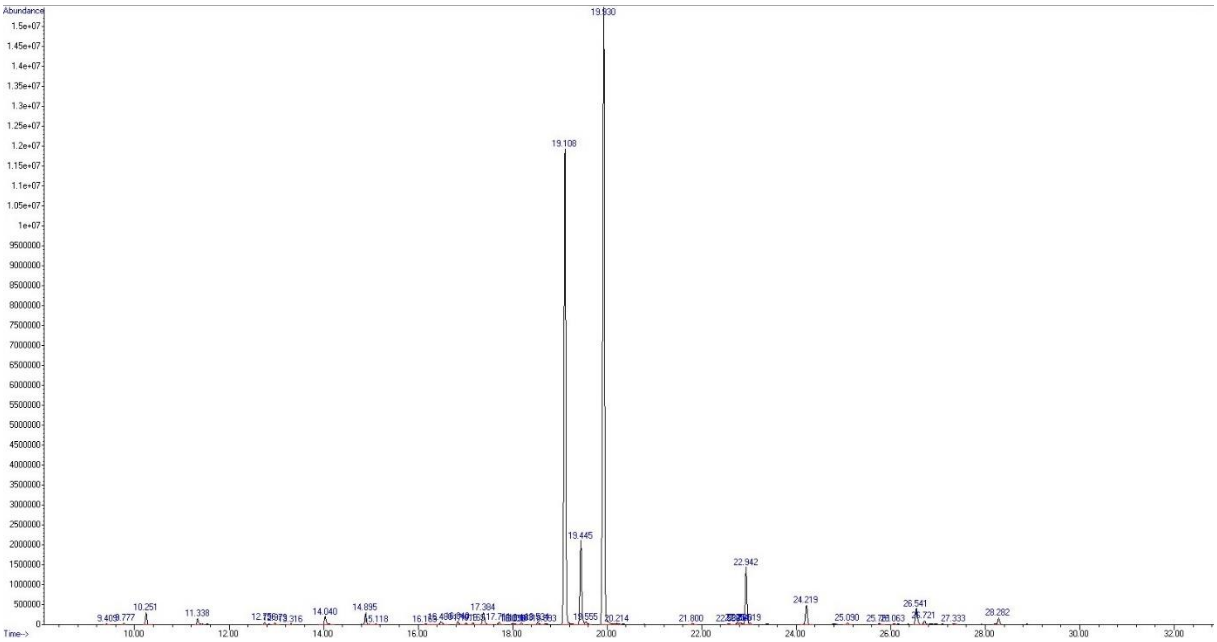

**Melaleuca**

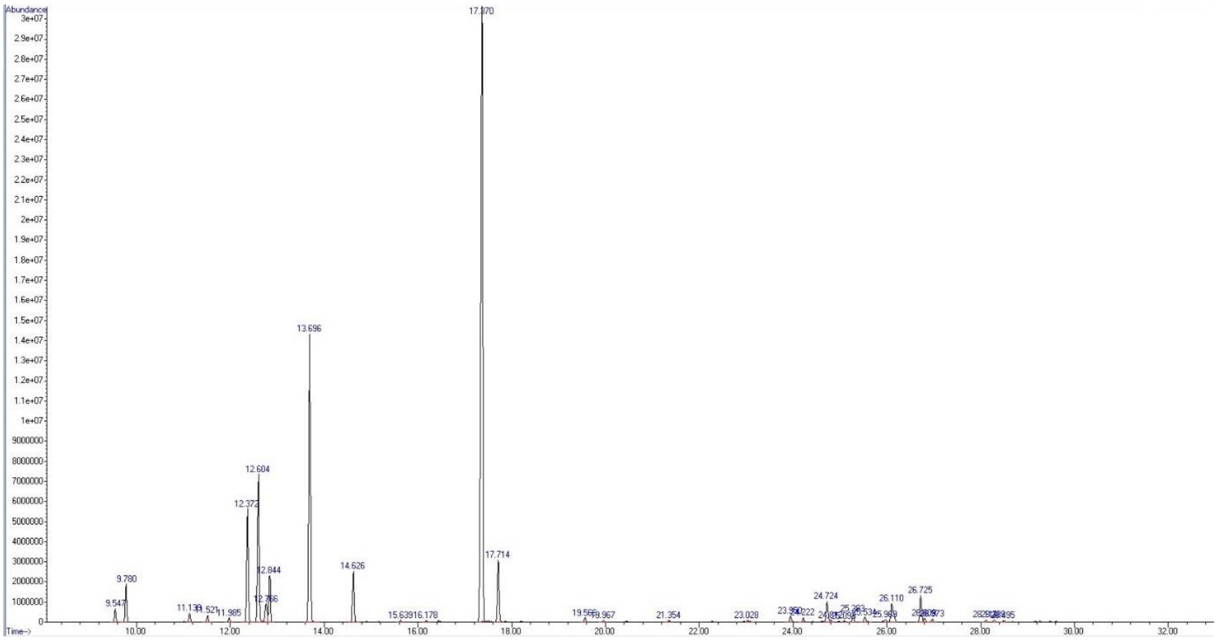


**Eucalyptus**

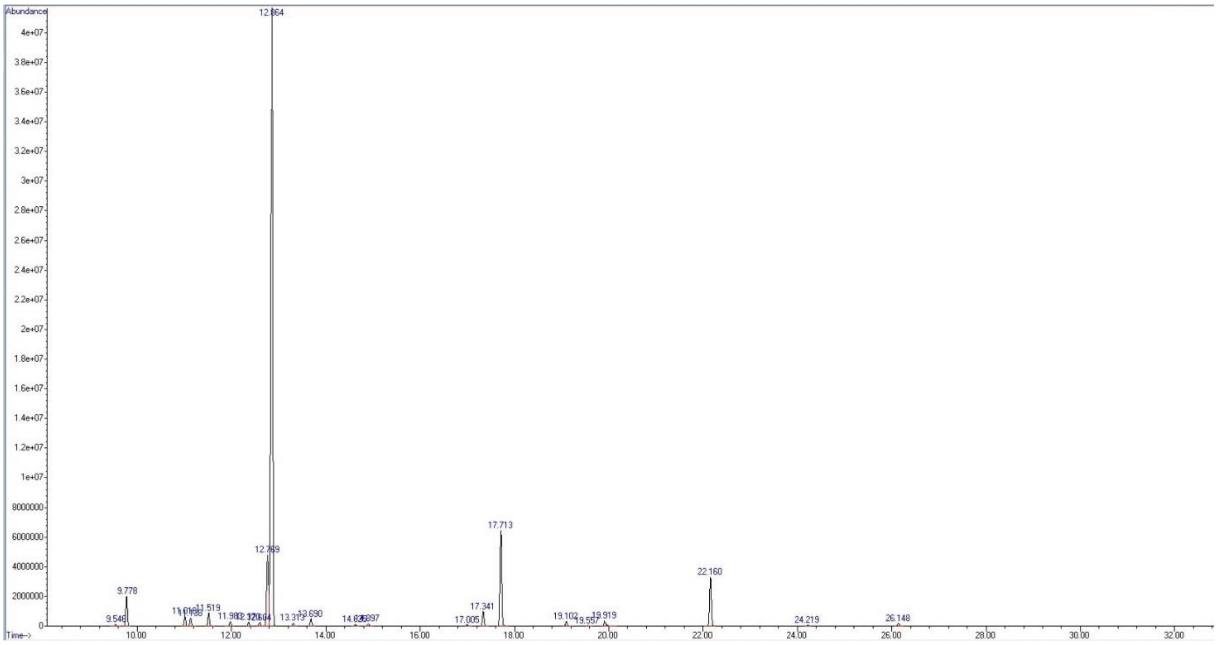

**Lavender**

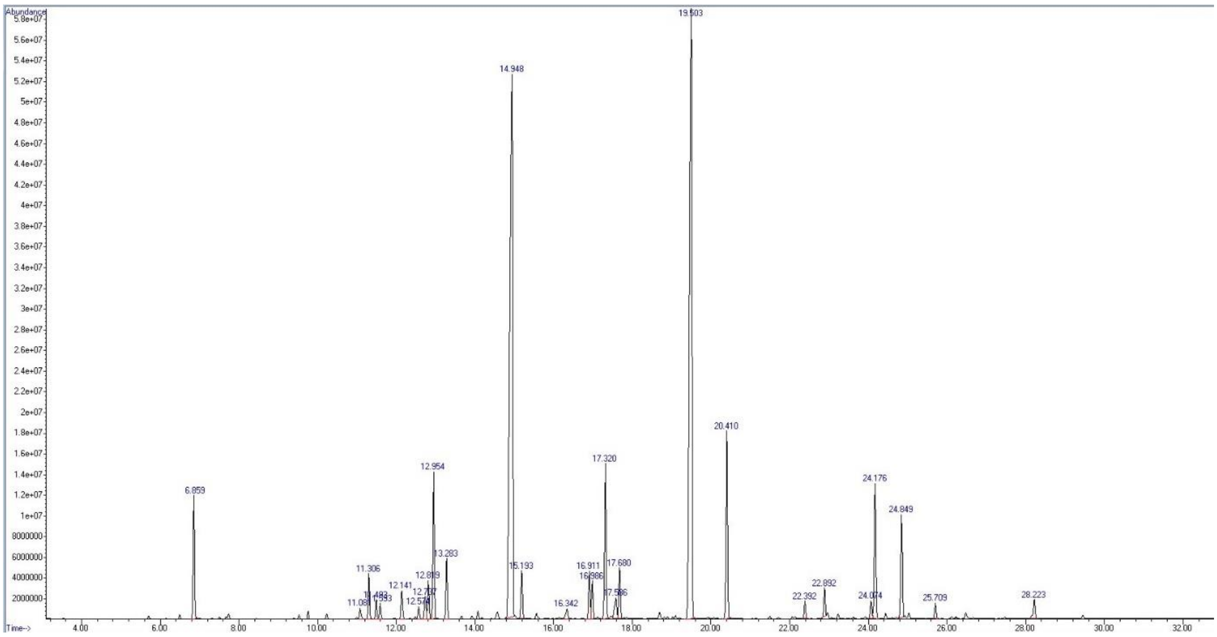


**Clary sage**

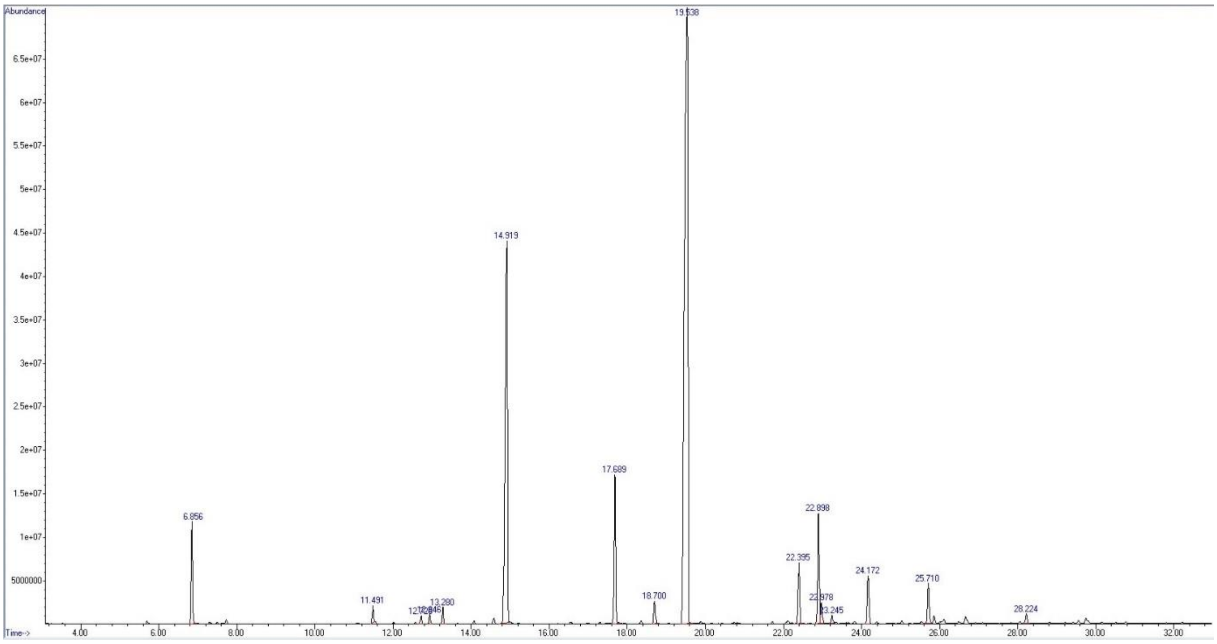

Supplement: Supplementary file 1 [file molecules-24-04570-s001.pdf]
